# Supplementary material for: Large Language Models and Genomics for Summarizing the Role of microRNA in Regulating mRNA Expression
Source: Biomedicines. 2024 Jul 10;12(7):1535. doi: 10.3390/biomedicines12071535 (PMC11274411; doi:10.3390/biomedicines12071535)
Supplement: Supplementary file 1 [file biomedicines-12-01535-s001.zip › Supplementary Data S1.pdf]

Annotation Guidelines

m-RNA / Gene – miRNA interaction

The document includes two sets of annotation rules.

1. Rules related to entities, gene/m-RNA and miRNA
2. Rules related to relation between gene/m-RNA and miRNA

Rules related to entities are mandatory because we adopted a semi-automated annotated process. We used our existing scripts to tag the gene/m-RNA mentions. We utilized the regular expression in Python to tag the miRNA mentions. Our approach may miss tagging of certain entities and produce erroneous tagging. It is necessary to tag the entities that are not tagged and correct the erroneous tagging prior to annotating the relations.

1. Rules related to entities, gene/m-RNA and miRNA

Rule 1.1. The left and right boundaries of the annotated entities should be correct.

Example 1:

> Augmentation of CBX7 by knockdown of miR-182 expression, in turn, positively regulated the expression of E-cadherin, a key protein involved in maintaining normal epithelial cell morphology, which is commonly lost during neoplastic progression

This sentence contains two gene mentions, CBX7 and E-cadherin, and a mi-RNA, miR-182. The gene entity should be tagged between <Gene> and </Gene>. The mi-RNA entity should be tagged between <miRNA> and </miRNA>.

Annotated sentence:

Augmentation of <Gene>CBX7</Gene> by knockdown of <miRNA>miR-182</miRNA> expression, in turn, positively regulated the expression of E-cadherin, a key protein involved in maintaining normal epithelial cell morphology, which is commonly lost during neoplastic progression

A gene entity may include one or more words. The tagging must cover all the words related to the gene. It is possible that the left or right boundary of the gene or mi-RNA may miss certain words that should be a part of the entity or include additional words that should not be a part of the entity. These errors should be corrected prior to annotating the relations.

The possible errors are:

1A. The left most boundary may miss one or more preceding words that should be the part of the annotated entity.

Example 2:

Luciferase assay and transfection confirmed <miRNA>miR-143</miRNA> binding to 3' UTR of prostaglandin-<Gene>endoperoxidase synthase 2</Gene> (<Gene>PTGS2</Gene>) mRNA and <miRNA>miR-143</miRNA> regulation of <Gene>PTGS2</Gene> in AMCs.

Error: prostaglandin- should be included in the gene name.

Reannotated entity:

Luciferase assay and transfection confirmed <miRNA>miR-143</miRNA> binding to 3' UTR of <Gene>prostaglandin-endoperoxidase synthase 2</Gene> (<Gene>PTGS2</Gene>) mRNA and <miRNA>miR-143</miRNA> regulation of <Gene>PTGS2</Gene> in AMCs.

NOTE: This sentence includes multiple genes and miRNA. While annotating the relation, the annotators must consider Example 7 (Rule 2.1).

1B. The right most boundary may miss one or more succeeding words that should be the part of the annotated entity.

Example 3:

Furthermore, we identified <Gene>BCL2</Gene>-associated athanogene 3 (<Gene>BAG3</Gene>), an anti-apoptosis protein, to be a target of <miRNA>miR-345</miRNA>

Error: associated athanogene 3 is part of BCL2 and it is not tagged. It should be tagged together.

Reannotated entity:
Furthermore, we identified <Gene>BCL2-associated athanogene 3 </Gene> (<Gene>BAG3</Gene>), an anti-apoptosis protein, to be a target of <miRNA>miR-345</miRNA>

NOTE: Though BAG3 is an abbreviation of gene, BCL2-associated athanogene 3, they should be considered as independent entities for relation extraction. Based on this statement, the sentence is considered to include multiple genes. While annotating the relations, the annotators must follow Example 7 (Rule 2.1).

1C. The left most boundary may include one or more preceding words that should NOT be the part of the annotated entity.

Example 4:

<miRNA>miR-23A</miRNA> in amplified 19p13.13 loci <Gene>targets metallothionein 2A</Gene> and promotes growth in gastric cancer cells.

Error: targets is not part of metallothionein 2A. It should not be tagged together.

Reannotated entity:
<miRNA>miR-23A</miRNA> in amplified 19p13.13 loci targets <Gene>metallothionein 2A</Gene> and promotes growth in gastric cancer cells.

1D. The right most boundary may include one or more succeeding words that should **NOT** be the part of the annotated entity.

Example 5:

<miRNA>microRNA 146a</miRNA> expression in rheumatoid arthritis: association with <Gene>tumor necrosis factor-alpha</Gene> and disease activity.

Error: <Gene>tumor necrosis factor </Gene> is the right tag. The gene name (or the gene symbol) for tumor necrosis factor is TNF. TNF-alpha is an alias. There is no exact mention of tumor necrosis factor-alpha in Entrez gene. Therefore, we retag the entity as per the entries in Entrez gene.

Reannotated entity:
<miRNA>microRNA 146a</miRNA> expression in rheumatoid arthritis: association with <Gene>tumor necrosis factor</Gene>-alpha and disease activity.

Rule 1.2. The automated tagging process may miss to tag certain entities. Such entities must be tagged with the related tags.

Example 6:

Here, we used a computational approach to identify <miRNA>miR-22</miRNA>, <miRNA>miR-25</miRNA>, and miR-302 as three <Gene>PTEN</Gene>-targeting microRNA (miRNA) families found within nine genomic loci.

Error: miR-302 is not tagged but should be tagged.

Reannotated entity:
Here, we used a computational approach to identify <miRNA>miR-22</miRNA>, <miRNA>miR-25</miRNA>, and <miRNA>miR-302</miRNA> as three <Gene>PTEN</Gene>-targeting microRNA (miRNA) families found within nine genomic loci.

NOTE: This sentence includes multiple miRNA. While annotating the relation, the annotators must consider Example 7 (Rule 2.1).

2. Rules related to relation between gene/m-RNA and mi-RNA

The relation between a gene/m-RNA and a mi-RNA is marked with an appropriate label:

A. True, when the sentence conveys a relation between a gene/m-RNA and a mi-RNA. This includes two sub-labels:
    a. Positive, when the sentence conveys a positive relation between a gene/m-RNA and a mi-RNA
    b. Negative, when the sentence conveys a negative relation between a gene/m-RNA and a mi-RNA
B. False, when the sentence conveys no relation between a gene/m-RNA and a mi-RNA.

The annotators will give any one of the following labels for every sentence in the corpus: positive, negative, or false.

Rule 2.1. A sentence is labeled as "Positive" if it conveys a positive association between the annotated entities.

If there are multiple entities, the annotator should create instances of the sentence to represent only a pair of tagged gene/m-RNA and mi-RNA and annotate the instance based on the relation between the tagged gene/m-RNA and mi-RNA.

Example 7:

Our findings identify <miRNA>miR-96</miRNA> as a potent regulator of <Gene>KRAS</Gene>, which may provide a novel therapeutic strategy for treatment of pancreatic cancer and other <Gene>KRAS</Gene>-driven cancers.

The sentence includes two mentions of the same gene. The annotators should take two instances and annotate the label for each instance. An instance should include only one tagged gene/m-RNA and a tagged mi-RNA as shown below:

Instance 1:
Our findings identify <miRNA>miR-96</miRNA> as a potent regulator of <Gene>KRAS</Gene>, which may provide a novel therapeutic strategy for treatment of pancreatic cancer and other KRAS-driven cancers.
Label for instance 1: Positive

Instance 2:
Our findings identify <miRNA>miR-96</miRNA> as a potent regulator of KRAS, which may provide a novel therapeutic strategy for treatment of pancreatic cancer and other <Gene>KRAS</Gene>-driven cancers.
Label for instance 2: Positive

Rule 2.2. A sentence is labeled as “Negative” if it conveys a negative association between the annotated entities.

If there are multiple entities, the annotator should create instances of the sentence to represent only a pair of tagged gene/m-RNA and mi-RNA and annotate the instance based on the relation between the tagged gene/m-RNA and mi-RNA.

<u>Example 8:</u>

<Gene>ACVR1</Gene>, a therapeutic target of fibrodysplasia ossificans progressiva, is negatively regulated by <miRNA>miR-148A</miRNA>.

<u>Label for Example 8:</u> Negative

Rule 2.3. A sentence is labeled as “False”, if includes the annotated entities but doesn’t convey any association between them.

<u>Example 9:</u>
CONCLUSION: miRNA profiling of <miRNA>miR-675</miRNA> and <miRNA>miR-335</miRNA> helps in discriminating <Gene>ACCS</Gene> from ACAs.

As mentioned earlier (see Rule 2.1), the annotators should generate instances of the sentence to represent only one pair of tagged gene and mi-RNA per instance and annotate the label for the instance based on the relation between the tagged entities (see Example 7).

<u>Instance 1:</u>
CONCLUSION: miRNA profiling of <miRNA>miR-675</miRNA> and miR-335 helps in discriminating <Gene>ACCS</Gene> from ACAs.
<u>Label for Instance 1:</u> False

<u>Instance 2:</u>
CONCLUSION: miRNA profiling of miR-675 and <miRNA>miR-335</miRNA> helps in discriminating <Gene>ACCS</Gene> from ACAs.
<u>Label for Instance 2:</u> False

<u>Example 10:</u>
Knockdown of <Gene>ROCK1</Gene> reversed EMT resembling that of <miRNA>miR-148A</miRNA> overexpression.
<u>Label for Example 10:</u> False

Rule 2.4. This is a special rule for sentences with multiple gene/m-RNA or mi-RNA entities. As mentioned earlier (see Rule 2.1), the annotators should generate instances

of the sentence to represent only one pair of tagged gene and mi-RNA per instance and annotate the label for the instance based on the relation between the tagged entities (see Example 7).

Here, a more complicated example is provided.

Example 11:

<miRNA>miR-15B</miRNA>/16-2 modulates the <Gene>CCND2</Gene> (<Gene>cyclin D2</Gene>), <Gene>CCND1</Gene> (<Gene>cyclin D1</Gene>), and <Gene>IGF1R</Gene> (<Gene>insulin-like growth factor 1 receptor</Gene>) genes involved in proliferation and antiapoptotic pathways in mouse B cells.

Instance 1:
<miRNA>miR-15B</miRNA>/16-2 modulates the <Gene>CCND2</Gene> (cyclin D2), CCND1cyclin D1), and IGF1R (insulin-like growth factor 1 receptor) genes involved in proliferation and antiapoptotic pathways in mouse B cells.
Label for Instance 1: Positive

Instance 2:
<miRNA>miR-15B</miRNA>/16-2 modulates the CCND2 (<Gene>cyclin D2</Gene>), CCND1 (cyclin D1), and IGF1R (insulin-like growth factor 1 receptor) genes involved in proliferation and antiapoptotic pathways in mouse B cells.
Label for Instance 2: Positive

Instance 3:
<miRNA>miR-15B</miRNA>/16-2 modulates the CCND2 (cyclin D2), <Gene>CCND1</Gene> (cyclin D1), and IGF1R (insulin-like growth factor 1 receptor) genes involved in proliferation and antiapoptotic pathways in mouse B cells.
Label for Instance 3: Positive

Instance 4:
<miRNA>miR-15B</miRNA>/16-2 modulates the CCND2 (cyclin D2), CCND1 (<Gene>cyclin D1</Gene>), and IGF1R (insulin-like growth factor 1 receptor) genes involved in proliferation and antiapoptotic pathways in mouse B cells.
Label for Instance 4: Positive

Instance 5:
<miRNA>miR-15B</miRNA>/16-2 modulates the CCND2 (cyclin D2), CCND1 (cyclin D1), and <Gene>IGF1R</Gene> (insulin-like growth factor 1 receptor) genes involved in proliferation and antiapoptotic pathways in mouse B cells.
Label for Instance 5: Positive

Instance 6:

<miRNA>miR-15B</miRNA>/16-2 modulates the CCND2 (cyclin D2), CCND (cyclin D1), and IGF1R (<Gene>insulin-like growth factor 1 receptor</Gene>) genes involved in proliferation and antiapoptotic pathways in mouse B cells.
Label for Instance 6: Positive

Rule 2.5. In the current study, we are interested only in the relations between a gene/m-RNA and a mi-RNA. Relations other than this are omitted. Below is an example:

Example 12:

Augmentation of <Gene>CBX7</Gene> by knockdown of <miRNA>miR-182</miRNA> expression, in turn, positively regulated the expression of <Gene>E-cadherin</Gene>, a key protein involved in maintaining normal epithelial cell morphology, which is commonly lost during neoplastic progression.

Below are the two possible instances for Example 10:

Instance 1:
Augmentation of <Gene>CBX7</Gene> by knockdown of <miRNA>miR-182</miRNA> expression, in turn, positively regulated the expression of E-cadherin, a key protein involved in maintaining normal epithelial cell morphology, which is commonly lost during neoplastic progression.
Label for Instance 1: Positive

Instance 2:
Augmentation of CBX7 by knockdown of <miRNA>miR-182</miRNA> expression, in turn, positively regulated the expression of <Gene>E-cadherin</Gene>, a key protein involved in maintaining normal epithelial cell morphology, which is commonly lost during neoplastic progression.
Label for Instance 1: --

This instance is out of scope for the current study because it conveys the information between a complex (with relation between a gene CBX7 and miR-182) and a gene, E-cadherin.
