# Supplementary material for: Large Language Models and Genomics for Summarizing the Role of microRNA in Regulating mRNA Expression
Source: Biomedicines. 2024 Jul 10;12(7):1535. doi: 10.3390/biomedicines12071535 (PMC11274411; doi:10.3390/biomedicines12071535)
Supplement: Supplementary file 1 [file biomedicines-12-01535-s001.zip › Supplementary Data S2.pdf]

Supplementary Data

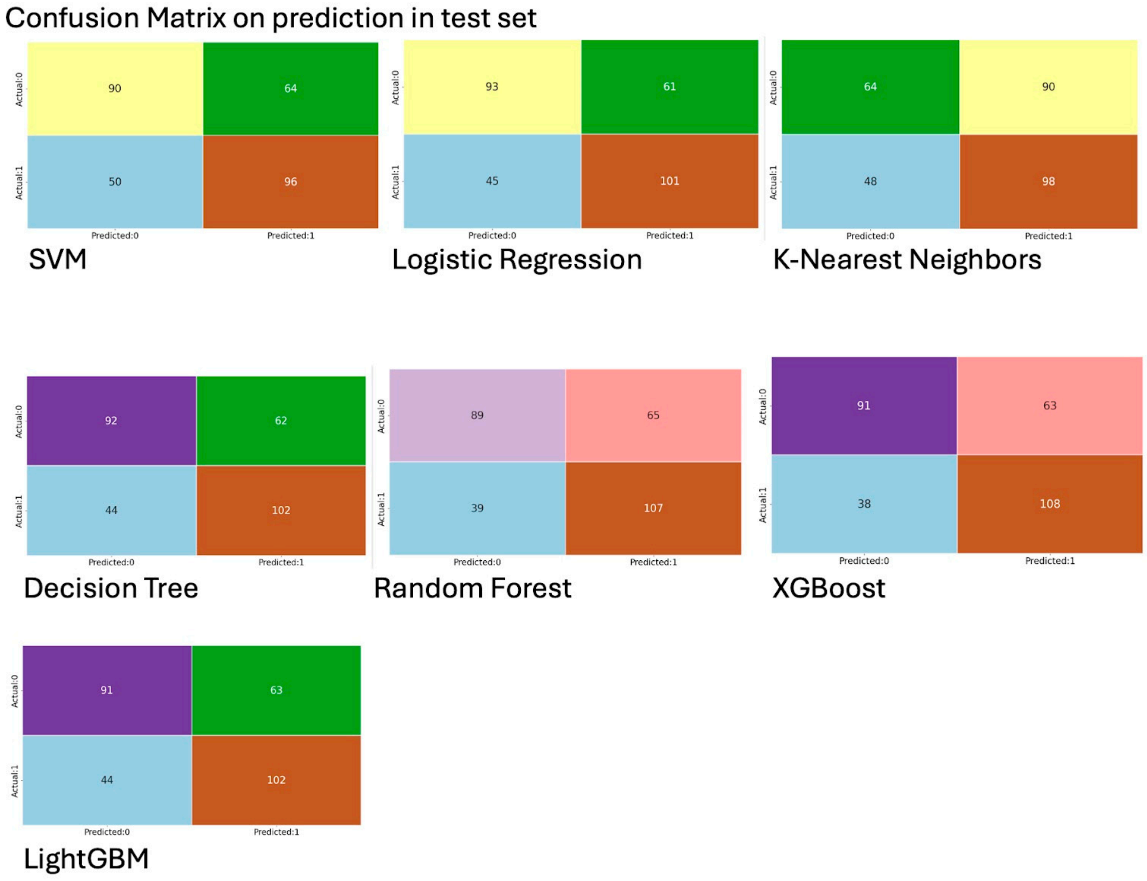

Figure S1: Confusion matrix on prediction on test set for the baseline machine learning models

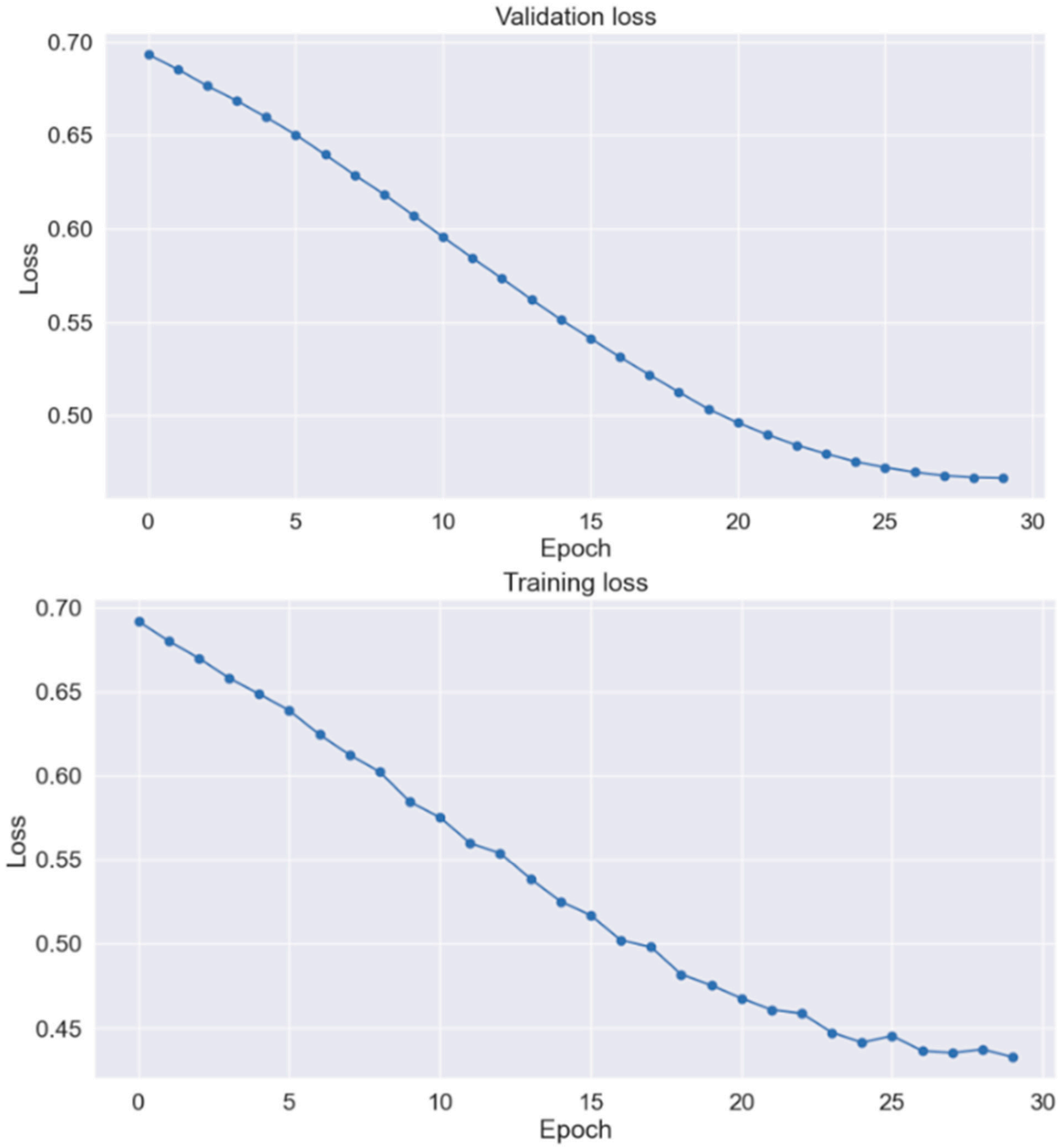

Figure S2: F-score on test dataset, validation loss, and training loss for PubMedBERT

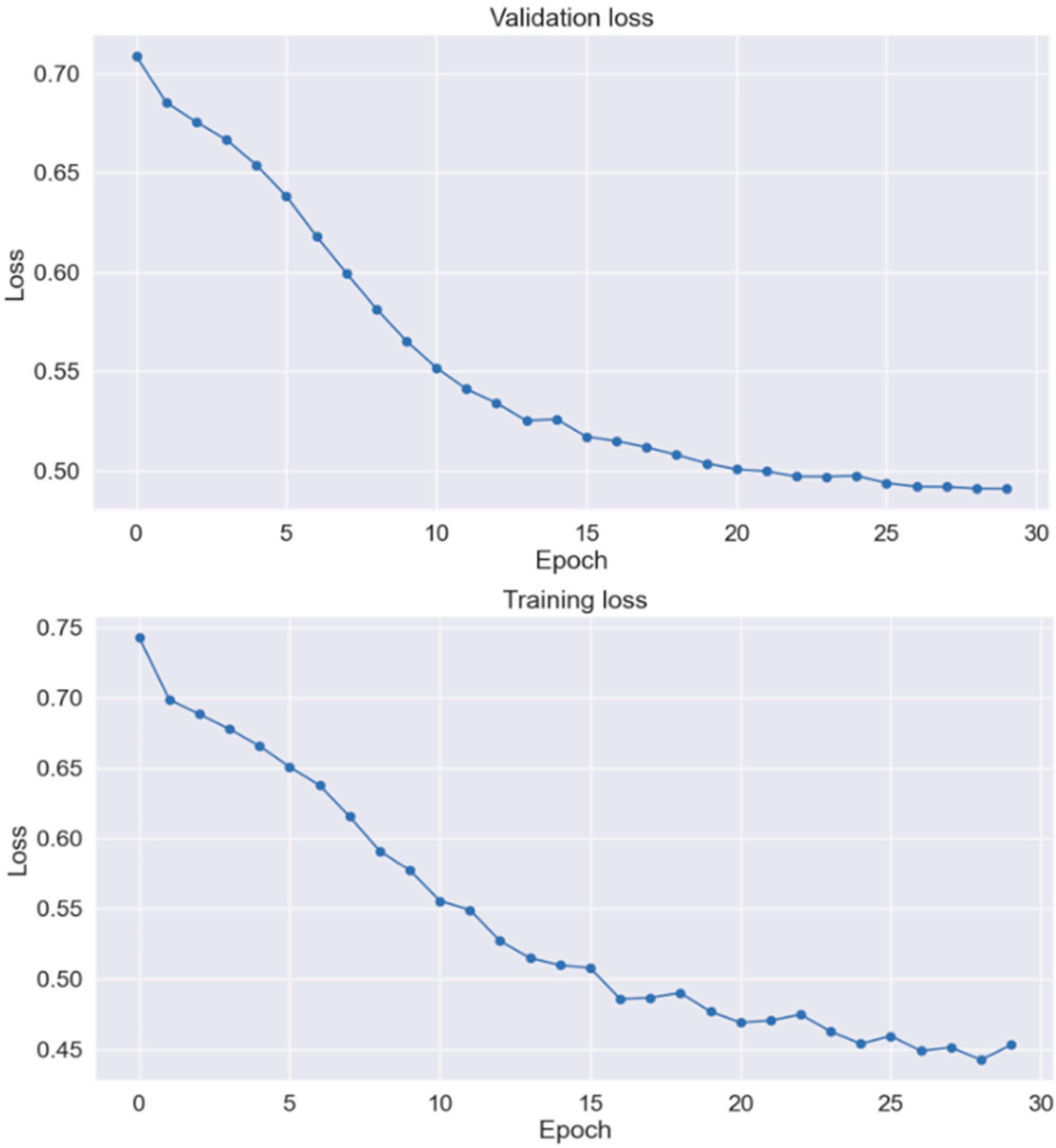

Figure S3: F1-score on test dataset, validation loss, and training loss for BioBERT

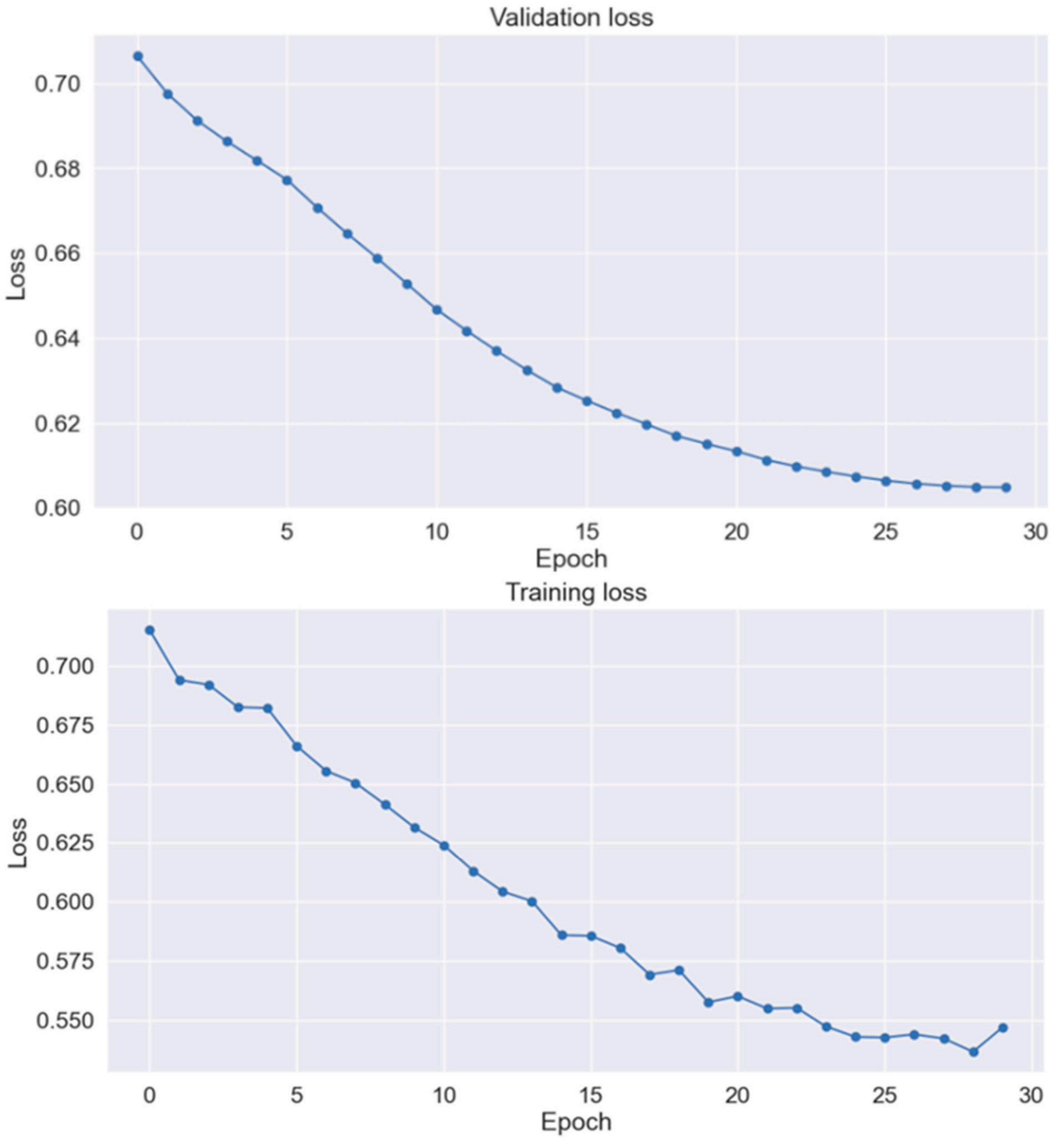

Figure S4: F1-score on test data, validation loss and training loss for ClinicalBERT

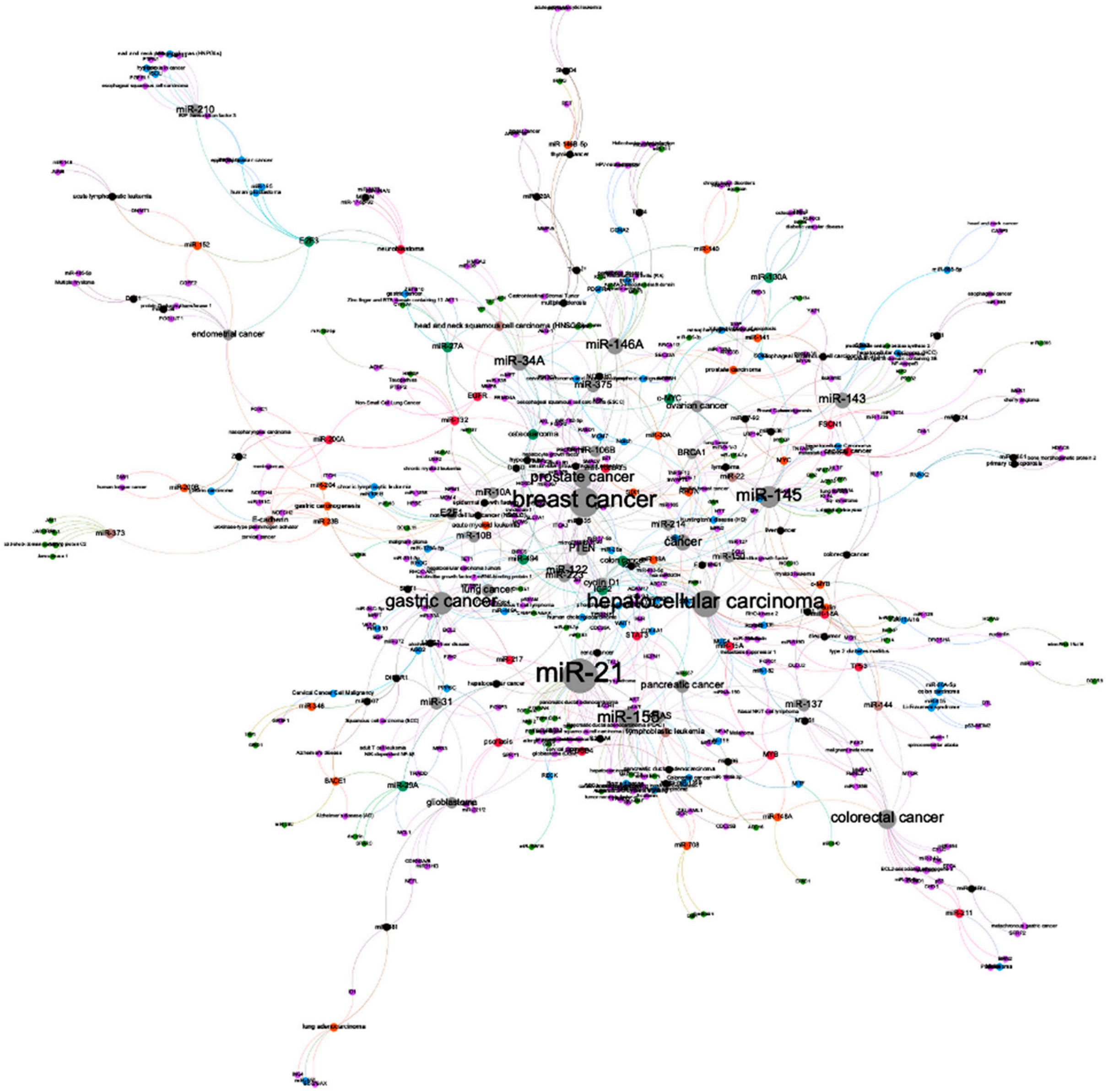

Figure S5: miRNA–mRNA–disease network from all the positive annotated relations.
